# Fetal Hepatic Circulation: From Vascular Physiology to Doppler Assessment

**DOI:** 10.3390/diagnostics15243147

**Published:** 2025-12-10

**Authors:** Inês Gil-Santos, Luís Guedes-Martins

**Affiliations:** 1 Instituto de Ciências Biomédicas Abel Salazar, University of Porto, 4050-313 Porto, Portugal; luis.guedes.martins@gmail.com; 2 Departamento da Mulher e da Medicina Reprodutiva, Centro Materno Infantil do Norte, Centro Hospitalar Universitário de Santo António, Largo Prof. Abel Salazar, 4099-001 Porto, Portugal; 3 Unidade de Investigação e Formação, Centro Materno Infantil do Norte, 4099-001 Porto, Portugal; 4 Instituto de Investigação e Inovação em Saúde, Universidade do Porto, 4200-319 Porto, Portugal

**Keywords:** Doppler, fetal hepatic circulation, hepatic artery

## Abstract

During fetal life, the hepatic artery (HA) is responsible for a small contribution to the total hepatic blood inflow; however, it plays a key role in maintaining liver perfusion and reflects fetal hemodynamic adaptation. With advances in ultrasonography, HA Doppler assessment has emerged as a potential tool for evaluating fetal well-being. This review aims to synthesize current knowledge on the embryology, anatomy, physiology, and Doppler assessment of the fetal hepatic artery, highlighting its diagnostic and clinical significance. A prenatal hepatic arterial buffer response (HABR), analogous to that in postnatal life, allows for compensatory vasodilatation when umbilical or portal venous inflow decreases. Doppler studies demonstrate that a reduced pulsatility index (PI) and resistance index (RI) and an increased peak systolic velocity (PSV) correspond to enhanced arterial flow and decreased vascular resistance. These patterns have been observed in fetal growth restriction (FGR) and certain chromosomal abnormalities. Fetal hepatic artery Doppler assessment contributes to the understanding of fetal adaptation to hypoxia and has a promising role in fetal well-being evaluation. As of now, there are no established reference curves, and it has not yet been incorporated into routine obstetric screening; future research should focus on standardizing measurement techniques and validating its prognostic value.

## 1. Introduction

The fetal liver is a central organ in intrauterine life, functioning not only as a site of hematopoiesis and metabolism but also as a key regulator of blood flow between the placenta and the systemic circulation; it actively modulates oxygen and nutrient distribution to ensure optimal growth and preferential perfusion of vital structures such as the brain and heart [1,2,3]. The cardiovascular system is the first to develop in the human embryo, with a detectable heartbeat by day 23 of embryogenesis, and the hepatic circulation soon emerges as a crucial component of this developing network [4,5,6].

The hepatic artery (HA) develops later, around the 8th week of gestation. During fetal life, it contributes a relatively minor portion of total hepatic blood flow—typically around 10%—compared with the umbilical and portal venous systems [7]. Nevertheless, the HA plays an increasingly recognized role in maintaining hepatic perfusion, especially under conditions of reduced venous inflow. Evidence suggests that a hepatic arterial buffer response (HABR), analogous to that existent in postnatal life, is already present prenatally, allowing compensatory arterial vasodilation when umbilical venous flow decreases, such as in case of ischemia/hypoxemia, and thus maintaining a constant hepatic blood flow [8,9,10].

Similar to the umbilical artery and other umbilical cord parameters such as abnormal umbilical coiling [11], advances in fetal Doppler ultrasonography have made it possible to visualize and quantify hepatic arterial flow in utero, offering a non-invasive window into hepatic perfusion and fetal hemodynamic regulation. Doppler parameters such as the pulsatility index (PI), resistance index (RI), and peak systolic velocity (PSV) in the fetal hepatic artery provide indirect markers of vascular resistance and compensatory blood flow mechanisms [12,13]. Reduced PI or RI and elevated PSV have been associated with adaptive responses to compromised venous return, as seen in fetal growth restriction (FGR) and other forms of placental insufficiency [12,14,15,16].

In this context, the hepatic artery has gained growing attention as a potentially sensitive indicator of fetal circulatory adaptation. Altered HA Doppler parameters have been reported in a variety of conditions, including fetal growth restriction (FGR), twin-to-twin transfusion syndrome (TTTS) and gestational diabetes [8,10,12,15,16,17,18,19]. Early gestational assessment of hepatic artery impedance may also hold promise in first-trimester screening for aneuploidies and congenital heart defects [16].

This review aims to provide a comprehensive overview of the current evidence regarding the fetal hepatic artery and its Doppler assessment in both low-risk and high-risk pregnancies. We hypothesize that evaluating impedance changes in the fetal hepatic artery may facilitate the early identification of high-risk pregnancies, as well as serve as a marker for chromosomal abnormalities, potentially as early as the first trimester.

## 2. Materials and Methods

PubMed and Medline databases were thoroughly searched using the mesh terms “fetal hepatic circulation”, “fetal vascular physiology”, “liver embryology”, “liver microcirculation”, “intrahepatic flow distribution”, “fetal hematopoietic microenvironment”, “liver blood flow regulation”, “Doppler ultrasonography” and “fetal hepatic artery”, considering the subject of each topic of study, between April 2025 and October 2025, by one author (I.G-S.).

Publications were assessed for inclusion by one author (I.G-S.) following predetermined criteria. Inclusion criteria included publications (articles, books, and guidelines) written in English, published until October 2025. Articles published as abstracts were included if information was adequate to assess inclusion criteria and data on relevant outcomes were reported. Opinion articles were excluded.

**Figure 1:**
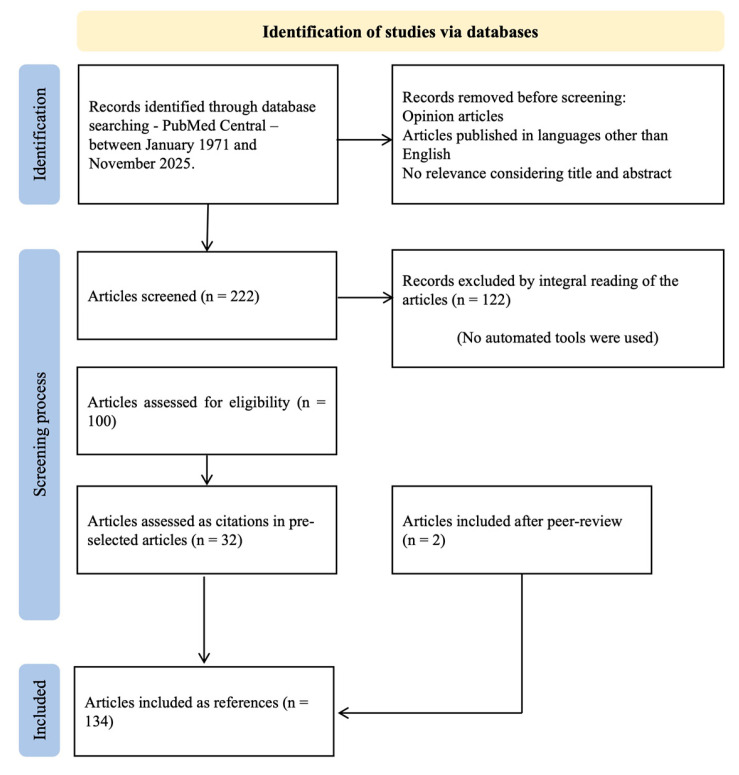
Literature search and selection process.

Two hundred and twenty-two articles were considered after reviewing the titles and abstracts of the articles, considering the relevance of the information for the intended review. Analysis of the selected sources implied an integral reading of the publications, and the authors extracted information on their results, discussion, and conclusion. Thirty-two articles were added as references suggested in the articles initially read, and two articles were added after suggestions from the reviewers during peer review. After their integral lecture, one hundred and twenty-two articles were excluded, as they were deemed not relevant to the subject. In the end, 134 articles were used as references in this bibliographic review (Figure 1).

This review focuses on the state of the art on fetal hepatic artery Doppler assessment, highlighting the lacunae in the field. We provide a structured overview of the fetal hepatic circulation, integrating embryological, anatomical, physiological, and Doppler ultrasound perspectives. By combining these perspectives, the review establishes a comprehensive framework for understanding fetal hepatic microcirculation and Doppler assessment parameters.

## 3. The Fetal Hepatic Circulation: Embryology and Anatomy

The fetal liver plays a central role in hematopoiesis and metabolism during gestation. Unlike in postnatal life, where the liver primarily functions as a metabolic and detoxifying organ, in the fetus, it is fundamental in blood flow regulation between the placenta and the systemic circulation [1,2,3].

The cardiovascular system is the first organ system to develop in the human embryo, with a detectable heartbeat by day 23 of development [4,5,6]. The hepatic circulation originates in early embryogenesis and becomes an essential component of the intrauterine cardiovascular system. The fetal liver is first identifiable at approximately 3–4 weeks of intrauterine development as a local endodermal thickening in the ventral wall of the distal foregut between the heart cranially and the yolk sac caudally [1,20,21,22].

During the 3rd week of gestation, paired vitelline and umbilical veins establish a primitive vascular plexus within the septum transversum, which subsequently integrates into the developing hepatic cords to form an extensive vascular network known as the hepatic sinusoids [4,23]. This network constitutes the first hepatic vascular bed, initially draining both yolk sac and placental blood. As development progresses, the vitelline veins contribute primarily to the formation of the portal venous system, whereas the umbilical veins become the dominant source of oxygenated blood to the liver [24,25]. By the 4th to 5th weeks, the right umbilical vein undergoes involution, leaving the left umbilical vein as the exclusive outlet for placental return [25].

During the 4th to 6th weeks of gestation, a shunt forms connecting the left umbilical vein—via the portal sinus—directly to the inferior vena cava (IVC): the ductus venosus (DV). This shunt serves to bypass the hepatic sinusoids and allows a portion of highly oxygenated placental blood to reach the systemic circulation, ensuring preferential perfusion of vital organs (such as the fetal heart and brain) [1,26].

The hepatic artery begins its development around the 8th week of gestation, arising from the common hepatic artery, itself a branch of the celiac trunk, one of the major anterior branches of the abdominal aorta. The common hepatic artery then gives rise to the proper hepatic artery, which subsequently divides into the right and left hepatic arteries, which supply the corresponding lobes of the liver. During fetal life, the hepatic artery plays a secondary role in hepatic perfusion, contributing with a relatively small proportion of total liver blood flow, typically less than 10%, compared to the umbilical and portal venous systems [7].

The left umbilical vein enters the liver at the hilum, where it forms the portal sinus. From the portal sinus, blood is distributed to the left and right portal branches, supplying the hepatic parenchyma, and to the DV, which provides a direct passage to the IVC. The DV arises from the portal sinus as a straight, branchless vessel, ascending toward the diaphragm and joining the IVC [27,28,29,30,31].

The extrahepatic portal vein drains into the portal sinus near the origin of the right intrahepatic portal vein. Numerous small hepatic veins converge into three main hepatic veins that drain into the subdiaphragmatic vestibulum [7]. The left hepatic lobe is supplied predominantly by umbilical venous blood, while the right lobe receives a mix of umbilical and portal venous blood [27,28]. At birth, the umbilical vein and ductus venosus close, and the portal vein becomes the sole afferent vessel to the liver [7,32].

As such, the fetal hepatic circulation serves dual, complementary functions: through the DV, it ensures rapid delivery of oxygenated blood to the heart and brain; through intrahepatic perfusion, it sustains hepatic development and function, including metabolic storage and hematopoiesis [33,34].

In the fetus, an average of 70% to 80% of the oxygenated umbilical venous return perfuses the liver tissue, while 20% to 30% bypasses the liver through the DV during the second half of pregnancy [35,36,37,38]. The proportion of blood that is shunted through the DV also varies under conditions of fetal compromise; in the presence of hypoxemia or fetal growth restriction (FGR), for instance, preferential DV shunting increases, reflecting an adaptive redistribution of cardiac output [39,40,41,42].

Given these implications, a detailed understanding of embryology and anatomy of the fetal hepatic circulation provides the foundation for the interpretation of DV and other hepatic vessels, Doppler velocimetry. Non-invasive tools such as these ones have become integral to the evaluation of fetal well-being, particularly in high-risk pregnancies, such as those complicated by FGR or congenital heart defects [28,43,44].

## 4. Microcirculation and Intrahepatic Flow Distribution

In early embryogenesis (4th–6th weeks of gestation), the liver primordium interacts with the vitelline and umbilical veins, forming the portal vein from vitelline segments and establishing the portal sinus and DV as key fetal vascular structures [1,23]. As hepatic cords invade the septum transversum, hepatic sinusoids emerge, initially displaying primitive capillary features and supporting hematopoiesis [1,45].

From the 10th to 25th weeks of gestation, angiogenesis and vasculogenesis drive the formation of intrahepatic arteries and capillaries, while portal veins and sinusoids undergo differentiation. Sinusoidal endothelial cells (SEC) undergo a two-step maturation process: first, they lose markers of continuous endothelium (including PECAM-1 and CD34 [46]) and reduce perisinusoidal laminin and fibronectin deposition, acquiring a fenestrated phenotype [45]. Second, they gain adult functional markers (e.g., stabilin-2, SE-1, ICAM-1, CD32, CD14), a process regulated by Vascular Endothelial Growth Factor (VEGF) and modulated by Transforming Growth Factor-β (TGF-β) signaling [45,47]. The acquisition of these markers is completed by 20 weeks of gestation, conferring adult-like functional properties to SEC [47].

During this period, there is maximal expression of VEGF and its receptors, reflecting active vascular remodeling [48,49]. VEGF, secreted by developing bile ducts and hepatoblasts, establishes a gradient that guides endothelial cell migration and proliferation. Endothelial precursors express the Vascular Endothelial Growth Factor Receptor-2 (VEGFR-2) (Flk-1/KDR), which mediates VEGF-induced signaling essential for vessel sprouting, branching, and maturation [46,50,51]

Angiopoietins and their receptor Tie-2 are also critical for vascular maturation and remodeling. Angiopoietin-1, produced by hepatoblasts, promotes vessel stabilization and maturation, while Angiopoietin-2, expressed by mural cells, modulates vessel plasticity and destabilization, facilitating VEGF-driven angiogenic sprouting. The reciprocal expression of these factors and their receptors is essential for hepatic artery development and remodeling in close association with bile duct morphogenesis [52,53].

In the fetal liver, the umbilical vein (UV) is the main afferent vessel, which delivers oxygen and nutrient-rich blood from the placenta. To a lesser extent, the liver also receives inflow from the portal vein (PV), which carries blood from the gastrointestinal tract and splanchnic circulation [27], and a minor contribution from the fetal hepatic artery [7].

The intrahepatic flow distribution is characterized by a significant proportion of umbilical venous blood bypassing the hepatic sinusoids via the DV and entering the IVC. This shunt may account for up to 50% of umbilical blood flow in early gestation, decreasing to approximately 20% near term. However, wide variability has been reported, with values ranging from 15% to 40% depending on methodology and fetal condition. The remainder blood perfuses the hepatic microvasculature [27,35,54].

The interaction among the mother, placenta, and fetus is crucial, as disruptions in placental development can compromise the delivery of essential nutrients and oxygen to the fetus; as such, fetal growth and development are shaped by a complex interplay of multiple factors [55]. The intrahepatic flow distribution is highly organized: the left hepatic lobe is supplied almost exclusively by the UV, while the right lobe receives a mix of UV and PV blood. This creates a functional dichotomy, with the left lobe exposed to higher concentrations of placental nutrients (prioritized for growth and energy storage), and the right lobe integrating splanchnic-derived substrates [27,56,57]. In conditions such as FGR, the prioritization of UV flow to the left lobe becomes even more pronounced, with compensatory increases in PV flow to the right lobe, further accentuating the difference in substrate exposure between lobes [9,58,59].

The distribution of blood flow is directed by the relative vascular resistances of the hepatic microvasculature and the DV, as well as by dynamic regulatory mechanisms that respond to fetal metabolic needs and hemodynamic status [60,61]. For example, increased DV shunting occurs in response to fetal hypoxia, prioritizing oxygen and glucose delivery to vital organs, such as the brain and heart, at the expense of hepatic perfusion [28,62]. More so, altered intrahepatic flow distribution has been associated with adverse outcomes in FGR, where preferential DV shunting is regarded as part of the fetal ‘brain-sparing’ response [43,63,64,65,66].

At birth, closure of the umbilical vein and DV shifts all portal inflow to the portal vein, and the hepatic artery matures, extending branches throughout the liver. Postnatally, the sinusoidal network acquires its definitive fenestrated structure, supporting efficient exchange and metabolic functions. In adulthood, hepatic microcirculation is defined by mature sinusoids, regulated flow from the portal vein and hepatic artery, and a hierarchical lobular organization that ensures optimal intrahepatic flow distribution and metabolic zonation [67,68].

## 5. Hematopoietic Microenvironment in the Fetal Liver

Pluripotent hematopoietic stem cells (HSCs) give rise to lineage-specific progenitors that subsequently mature into the diverse blood cell types, including erythrocytes, megakaryocytes, lymphocytes, neutrophils, and macrophages, through the biologic process of hematopoiesis [69,70,71]. Fetal hematopoiesis takes place in the liver before colonization of the bone marrow, where it will persist into post-natal life [72,73,74].

The key features of hematopoiesis in the fetal liver include rapid expansion and differentiation of hematopoietic stem and progenitor cells (HSPCs), supported by a transient and highly specialized microenvironment [75,76,77], while the processes of self-renewal and differentiation are tightly regulated to preserve HSC homeostasis in the adult bone marrow [78,79,80]. The fetal liver serves as the principal site of hematopoiesis during mid-gestation, enabling both self-renewal and lineage commitment of HSPCs to meet the demands of fetal growth [76,77].

The hematopoietic microenvironment in the fetal liver is a specialized, transient niche that supports the rapid expansion and differentiation of HSPCs during embryonic development [76,81,82]. Key contributors include hepatoblasts, liver sinusoidal endothelial cells (LSECs), mesenchymal and mesothelial populations, as well as resident macrophages [83,84].

Hepatoblasts are a central component, providing key hematopoietic growth factors such as erythropoietin (EPO) and stem cell factor (SCF), which are critical for erythropoiesis and HSC maintenance [83,85].

The vascular organization and blood flow within the fetal liver are also central determinants of its hematopoietic microenvironment: portal vessels branching from the umbilical vein establish periportal zones enriched with Nestin+/NG2+ pericytes that foster stem cell expansion and maintenance [83,86]. Macrophages, especially yolk sac-derived subpopulations, regulate both erythropoiesis and granulopoiesis, and interact directly with HSCs to influence lineage fate [87].

Emerging evidence also suggests that epithelial-to-mesenchymal transition (EMT)-like processes in hepatic stromal cells provide additional regulatory inputs into niche remodeling [88]. Spatial mapping reveals that different hematopoietic progenitors have distinct preferences for neighboring stromal cells and regional localization, with the sub-mesothelium playing a prominent role in early hematopoiesis [83].

At a molecular level, the fetal liver niche is enriched for Wnt signaling regulators, which are implicated in supporting HSC proliferation and self-renewal, distinguishing it from the Notch-dominated adult bone marrow niche [89,90]. This dynamic and developmentally regulated microenvironment enables hematopoietic expansion before the transition to bone marrow hematopoiesis later in gestation [75,91].

As gestation advances and definitive hematopoiesis becomes established in the bone marrow, the fetal liver undergoes significant structural and functional changes. One of the key events is the closure of the umbilical vein inlet and the concomitant remodeling of the portal vascular network. These vascular transformations alter the hepatic microenvironment, leading to a decline in the signals necessary for HSC maintenance and proliferation. Consequently, HSCs progressively migrate from the liver to the developing bone marrow, which becomes the primary site of hematopoiesis in late gestation and postnatal life. This transition marks a crucial developmental shift from a transient hematopoietic organ to one primarily involved in metabolic and immunological functions [72].

## 6. Hepatic Arterial and Venous Blood Flow Regulation

In adult life, hepatic blood flow is characterized by a dual inflow system: approximately 75% of hepatic blood flow is supplied by the portal vein and the remaining 25% by the hepatic artery [1,92]. A key regulatory mechanism, the hepatic arterial buffer response (HABR), allows the hepatic artery to adjust its flow inversely to changes in portal vein inflow [30,93,94,95].

Adenosine plays a central role in the regulation of hepatic blood flow, specifically as the mediator of the HABR. Adenosine is continuously produced and released into the space of Mall (periportal space), surrounding terminal branches of the hepatic artery and portal vein. Its local concentration is primarily regulated by washout into the portal circulation. When portal venous flow decreases, adenosine washout is reduced, leading to its accumulation and subsequent vasodilation of the hepatic artery, thereby increasing arterial inflow to compensate for the reduced portal supply and maintaining overall hepatic perfusion [93,96,97].

**Figure 2:**
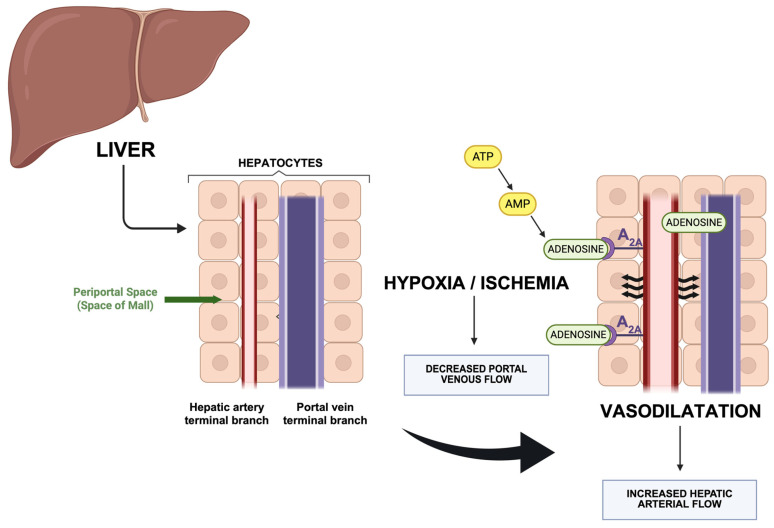
Schematic illustration of the Hepatic Arterial Buffer Response (HABR). Decreased portal venous flow resulting from hypoxia and/or ischemia leads to adenosine secretion into the space of Mall (through the breakdown of adenosine triphosphate (ATP) and adenosine monophosphate (AMP), which subsequently leads to hepatic arteriolar vasodilation via A_2A_ adenosine receptors. This results in an increase in hepatic arterial flow, thus maintaining overall hepatic perfusion [65,66,67,68,69,70,71]. Created in BioRender. Santos, I. (2025) https://BioRender.com/v560wat (accessed on 8 December 2025).

The vasodilatory effect of adenosine on the hepatic artery is mediated predominantly via A_2A_ adenosine receptors on vascular smooth muscle cells, which cause relaxation of hepatic arterial smooth muscle, and thus result in decreased vascular resistance and increased arterial flow [98,99,100,101] (Figure 2). This mechanism is consistent with adenosine’s broader role in vascular tone regulation throughout the body, especially under conditions of hypoxia or ischemia, where adenosine production is upregulated [102,103].

Adenosine is produced both intracellularly and extracellularly in the liver, primarily through the breakdown of adenosine triphosphate (ATP) and adenosine monophosphate (AMP) by ectonucleotidases CD39 and CD73 on the cell surface, as well as cytoplasmic 5′-nucleotidases within hepatocytes [104,105,106]. Under stress—such as hypoxia and ischemia—ATP released from hepatocytes and other liver cells is sequentially dephosphorylated to AMP and then to adenosine [104,105]. Through this mechanism, total hepatic blood flow remains relatively constant, ensuring stable oxygen and nutrient delivery to the liver despite fluctuations in portal venous input [93,96]. This mechanism is intrinsic to the hepatic artery; the liver cannot directly regulate portal venous inflow, which is primarily determined by splanchnic hemodynamics and cardiac output [96,107].

In the fetus, hepatic venous blood flow is primarily regulated by the distribution of umbilical vein blood between the liver and the DV, with no direct portal venous inflow as observed in the adult. The umbilical vein delivers oxygenated, nutrient-rich blood from the placenta, a portion of which enters the liver, while the remainder is shunted via the DV directly to the IVC [54,56].

The relative proportion of blood shunted through the DV versus that perfusing the liver is dynamically regulated by local vascular impedance, vessel anatomy, and physiological demands, including oxygen requirements and fetal distress [12,54,56,108,109]. Enhanced DV shunting occurs in response to hypoxia or FGR, thereby prioritizing the delivery of oxygen and nutrients to vital organs such as the brain and heart [54,56,60,110].

As previously stated, fetal hepatic arterial flow contribution is relatively minor compared to the umbilical venous supply. Recent evidence demonstrates that a hepatic arterial buffer response is active prenatally, with hepatic artery dilation in response to reduced umbilical venous flow to the liver, analogous to the adult mechanism, but with less pronounced magnitude in the fetus [8,9,10]. This response is mediated by changes in hepatic arterial resistance, likely also involving local vasoactive mediators such as adenosine [8].

Doppler studies in human fetuses have demonstrated that the hepatic artery pulsatility index (PI) decreases—reflecting vasodilation and increased flow—when umbilical venous flow is reduced, supporting the presence of this buffer response prenatally [8]. Findings suggestive of this HABR have also been documented in specific fetal conditions, such as twin-to-twin transfusion syndrome, where it is particularly pronounced in donor fetuses experiencing relative hypovolemia [17,18].

## 7. Hepatic Artery Doppler

Under normal conditions, approximately 75–90% of the fetal liver’s blood supply derives from the portal vein (PV), while the hepatic artery (HA) contributes the remaining 10–25% [92,111]. The HABR allows for the maintenance of hepatic perfusion under stress (hypoxemia/ischemia) by allowing the HA to adjust its flow inversely to changes in PV inflow [30,93,112]. Due to this mechanism, some studies have referred to the liver as a ‘fourth preferential organ’ (other than the heart, brain, and adrenal glands) [46,113,114].

In recent years, prenatal diagnosis has undergone continuous and dynamic progress, leading to the development of increasingly refined tools for detecting fetal congenital anomalies, chromosomal abnormalities, and identifying pregnancies at risk for various forms of placental insufficiency [115,116,117,118,119,120]. Recent advances in fetal Doppler ultrasonography have made it possible to visualize and quantify HA flow in utero, offering new insights into hepatic perfusion and fetal hemodynamic regulation. Doppler ultrasound parameters such as pulsatility index (PI), resistance index (RI), and peak systolic velocity (PSV) in the fetal HA serve as indirect indicators of hepatic perfusion dynamics and overall fetal well-being by reflecting changes in vascular resistance and compensatory blood flow mechanisms [8,13].

Lower PI and RI values indicate decreased vascular resistance, which is typically seen when hepatic arterial flow increases to compensate for reduced portal venous perfusion (as in the HABR mechanism) [8]. The first report on low-resistance HA flow in the fetus dates back to 1999, after it was documented in growth-restricted fetuses in the second trimester of pregnancy [14]. Elevated PSV in the hepatic artery may also signal increased compensatory arterial flow, and abnormal values (lower PI, higher PSV) have been associated with adverse pregnancy outcomes such as chromosomal abnormalities and congenital heart defects (CHD), suggesting that these Doppler indices may serve as early markers of fetal compromise [15,16].

As gestational age progresses, the fetal HA exhibits a progressive decline in both PI and RI, accompanied by an increase in PSV. This pattern reflects the physiological reduction in vascular resistance and increased blood flow to the fetal liver as gestation advances. Longitudinal Doppler studies demonstrated that PI and RI values are significantly lower in the third trimester compared to earlier stages, indicating gradual vasodilation and adaptation to the growing metabolic demands of the fetus. These changes parallel the decrease in impedance observed in other fetal abdominal arteries throughout pregnancy [8].

In first-trimester fetuses, the HA arises from the celiac artery—a branch of the aorta—and courses superoanteriorly towards the DV, with which it establishes close anatomical proximity [112].

**Figure 3:**
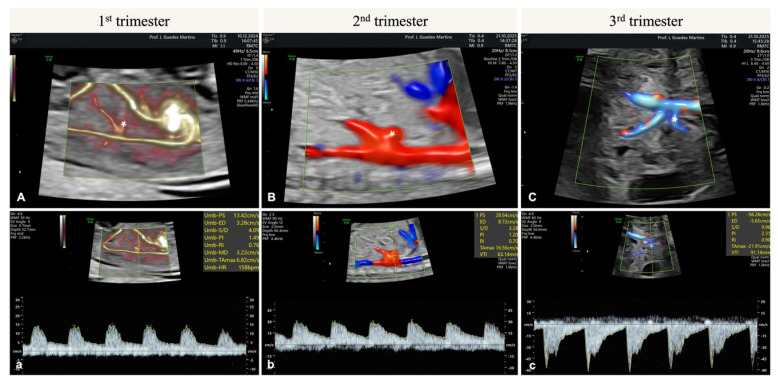
Fetal Hepatic Artery Doppler assessment during pregnancy. From left to right, the figure shows the Hepatic Artery (identified with a *) and its Doppler assessment showing normal velocity waveforms in the first (**A**,**a**), second (**B**,**b**) and third (**C**,**c**) trimesters. The HA (*) arises from the celiac artery—a branch of the abdominal aorta—and courses superoanteriorly towards the DV, with which it establishes close anatomical proximity. Throughout gestation, there is a decrease in both PI and RI, accompanied by an increase in PSV. This pattern reflects the physiological reduction in vascular resistance and increased blood flow to the fetal liver with progressive gestational age, paralleling the decrease in impedance observed in other fetal abdominal arteries throughout pregnancy.

Fetal HA Doppler ultrasound is ideally performed using a high-resolution ultrasound system with color and pulsed-wave Doppler. The examination is typically conducted with a transabdominal probe, using the mid-sagittal plane of the fetal abdomen. Doppler image should be magnified until the fetal abdomen occupies the screen. The HA is identified using color Doppler—most commonly by visualizing its left branch as it courses near the portal vein within the fetal liver (Figure 3); the sample volume width should be of 0.7—1.0 mm (similarly to DV Doppler study protocols), which should be placed in the hepatic artery, near the ductus venosus, to analyze its left branch. Doppler scale should be set low (<3 Hz) in order to visualize low-flow velocities, and the angle of insonation minimized (ideally <30°) to optimize velocity measurements and reduce error [8,16,19]. Waveforms should be recorded during fetal rest, avoiding fetal and maternal breathing/movement, and measurements should be averaged over at least three consecutive cardiac cycles (Figure 3) [16,19,121]. The fetal HA’s pulsatility index (HA-PI) and peak systolic velocity (HA-PSV) should be measured to determine HA flow [10,16]. Reporting results alongside umbilical artery (UA), middle cerebral artery (MCA) and ductus venosus (DV) indices might support a systemic view of hepatic/central circulation and fetal well-being [8,16,122].

Available literature indicates that Doppler assessment of the fetal HA may provide valuable information in both normal and high-risk pregnancies. Changes in hepatic artery pulsatility and flow velocity may reflect fetal stress, altered oxygenation, or chromosomal abnormalities, and they may serve as a predictive marker in these situations [15,16,123].

First-trimester applications of fetal hepatic artery Doppler ultrasound focus on early risk stratification. Measurement of HA-PI and PSV between 11 weeks and 13 weeks + 6 days can potentially help predict adverse pregnancy outcomes, particularly chromosomal abnormalities and CHD. Abnormal values (lower HA-PI and higher HA-PSV) are associated with increased risk, making this modality a useful adjunct to first-trimester screening protocols for fetal anomalies [16] (e.g., HA resistance appears to be reduced and blood flow increased in fetuses with Trisomy 21) [10].

When it comes to the second and third trimesters of pregnancy, the indications and clinical applications of fetal HA Doppler ultrasound are primarily investigational. In high-risk pregnancies, HA Doppler may be used to study perfusion changes in cases of suspected FGR, as altered hepatic perfusion dynamics can reflect compensatory mechanisms in response to placental insufficiency or other hemodynamic stressors [8,10,17,18]. Recent evidence shows that assessment of fetal hepatic perfusion in pregnancies complicated by gestational diabetes mellitus concluded that altered HA Doppler parameters also correlate with maternal glycemic control and may reflect fetal metabolic adaptation [19]. Other parameters, such as fetal liver volume, also appear to be correlated with maternal glycemic control [124,125] and fetal growth patterns, such as FGR [19,126,127,128].

Another application of hepatic artery Doppler pertains to twin-to-twin transfusion syndrome (TTTS), in which the donor twin experiences hypovolemia and reduced portal venous flow, which triggers HABR—manifested as increased HA-PSV—to maintain hepatic perfusion. This response is quantifiable by Doppler and is significantly elevated in donor twins compared to normal monochorionic diamniotic twins. After successful fetoscopic laser ablation of placental anastomoses, HA-PSV in donor twins decreases, indicating resolution of the compensatory response and improved hemodynamic status [17,18]. HA Doppler can serve as an adjunct marker of hemodynamic compromise in TTTS, reflecting the severity of circulatory imbalance and the effectiveness of therapeutic intervention. However, HA Doppler is not currently included in routine TTTS staging or management algorithms (which currently prioritize amniotic fluid assessment, bladder visualization, and Doppler studies of the UA and DV) [129,130,131].

To summarize, a decrease in hepatic artery pulsatility or resistance indices (HA-PI/RI), often accompanied by an increase in PSV, reflects reduced hepatic arterial resistance and a compensatory rise in arterial flow, typically occurring when umbilical or portal venous (UV/PV) inflow is diminished—corresponding to vasodilation/activation of the HABR, especially when DV-PI is high [8]. Conversely, higher HA-PI values have been observed in fetuses with abundant UV/PV inflow, as documented in normal reference cohorts [8]. These Doppler changes represent physiological adaptations rather than pathological findings, providing complementary insight into fetal hemodynamic regulation. Clinically, hepatic artery Doppler serves to contextualize fetal hemodynamics/circulatory compensation—particularly in conditions such as FGR or TTTS—and to enhance early risk assessment in the first trimester, while not supplanting established Doppler parameters such as for the UA, MCA or DV indices [132,133,134]. Current evidence is insufficient to support routine clinical use; findings should be interpreted as exploratory.

## 8. Discussion

The fetal hepatic circulation represents a dynamic and multifunctional system that plays a central role in both fetal development and adaptation to intrauterine conditions. It is uniquely structured to balance the competing demands of hepatic growth, hematopoiesis, and preferential oxygen delivery to vital organs such as the heart and brain. Understanding its embryological development, vascular organization, and hemodynamic regulation provides a crucial framework for interpreting Doppler findings and for identifying fetal compromise in clinical practice.

The hepatic artery begins its development around week eight of gestation, arising from the common hepatic artery—a branch of the celiac trunk, one of the major anterior branches of the abdominal aorta—and plays a secondary role in hepatic perfusion during fetal life, contributing with about 10% of inflow, a small contribution compared to the umbilical and portal venous systems [7].

The intrahepatic flow distribution reflects a finely tuned balance between the resistances of the hepatic microvasculature and the DV [60,61]. This balance ensures appropriate perfusion of the hepatic tissue while allowing adaptive redistribution in response to physiological stress. In pregnancies complicated by FGR, for instance, increased DV shunting and reduced hepatic perfusion exemplify the fetal “brain-sparing” phenomenon [43,63,64,65,66]. Postnatally, closure of the umbilical vein and DV marks the transition to a fully portal-dependent hepatic circulation, coinciding with the maturation of hepatic arterial branches and establishment of definitive sinusoidal networks [67,68].

With respect to hepatic blood flow regulation, the hepatic arterial buffer response (HABR) is a key mechanism ensuring perfusion stability. In adult life, this response compensates for fluctuations in portal inflow by inversely modulating hepatic arterial flow through adenosine-mediated vasodilation [93,94,95,96,97,98,99,100,101], preserving hepatic oxygen delivery under conditions of reduced portal perfusion [96,97,98,102,103]. This autoregulatory system maintains hepatic blood flow constancy, despite variations in splanchnic and systemic hemodynamics [96,107].

A similar but less pronounced mechanism has been described in the fetus. The distribution of umbilical venous blood between the liver and the DV is regulated by vascular impedance and physiological demands [12,54,56,108,109]. Under hypoxic stress, DV shunting increases while hepatic arterial resistance decreases, suggesting the presence of a fetal form of HABR [8,9,10]. Doppler studies confirm that a decline in hepatic artery PI accompanies reduced umbilical venous flow, indicating compensatory arterial dilation [8]. This adaptive mechanism underlines the ability of the fetal liver to modulate perfusion and sustain oxygenation when placental or venous inputs are compromised.

Fetal hepatic artery Doppler ultrasonography has emerged as a valuable, non-invasive tool for assessing these regulatory dynamics. Doppler parameters such as PI, RI, and PSV provide indirect measures of hepatic vascular resistance and flow compensation [8,13]. Lower PI/RI and higher PSV values are characteristic of enhanced hepatic arterial flow, typically observed when venous inflow is diminished, reflecting activation of the HABR, such as in cases of ischemia/hypoxemia [8,16].

Throughout gestation, there seems to be a physiological decrease in both HA-PI and HA-RI, accompanied by an increase in HA-PSV. This pattern reflects the physiological reduction in vascular resistance and increased blood flow to the fetal liver with progressive gestational age, which parallels the decrease in impedance that can be observed in other fetal abdominal arteries throughout pregnancy [8].

These Doppler indices have demonstrated clinical relevance across gestation, from association with chromosomal abnormalities in the first trimester—suggesting their potential role in early risk stratification [10,15,16]—to association with compensatory mechanisms in cases of placental insufficiency/FGR later on in pregnancy [8,10,17,18], proving it might be useful in fetal compromise detection/evaluation (e.g., to contextualize fetal hemodynamics/circulatory compensation in conditions such as FGR or TTTS, which might imply need for intervention/pregnancy termination).

In summary, the fetal hepatic circulation exemplifies the intricate interplay between developmental anatomy, vascular physiology, and adaptive hemodynamics. While HA Doppler is not yet part of standard screening protocols, its integration into comprehensive fetal assessment—particularly in high-risk pregnancies—offers promising diagnostic and prognostic value. Continued investigation will deepen our understanding of the fetal liver’s central role in regulating circulatory adaptation, offering insights into both physiological and pathological conditions [8,132,133,134].

However, despite its potential, reference data for normal Doppler parameters’ values remain limited, and there haven’t been established reference curves/charts. The few studies available are limited by small sample sizes, lack of standardization regarding the site of measurement, and unspecified gestational age intervals. Moreover, published reference ranges lack sufficient methodological rigor to allow for widespread clinical application. Another concern is the lack of intra- and inter-observer reproducibility studies on the fetal hepatic artery Doppler assessment, in order to validate its clinical applicability.

## 9. Future Research

Doppler assessment of the fetal hepatic artery (HA) has been shown to provide valuable insights into both normal and high-risk pregnancies. Its potential clinical applications include the early detection of chromosomal abnormalities and congenital heart defects, as well as the identification of placental insufficiency later in gestation. These findings emphasize the relevance of fetal HA Doppler as a promising tool for both diagnostic and prognostic purposes throughout pregnancy.

Establishing reference curves and percentile charts for fetal HA Doppler parameters across all trimesters is essential; such data would strengthen the clinical validity of this assessment, enabling its integration into routine obstetric ultrasound practice. In addition, standardized reference values would provide a foundation for comparative research into pathological conditions affecting fetal hemodynamics. Generating such data will require large prospective cohort studies.

Developing a standardized protocol to define normal impedance variations in the fetal hepatic artery throughout pregnancy—particularly during the first trimester—represents a crucial step towards establishing reliable reference curves for clinical application. To ensure reproducibility and broad applicability of these reference curves, several methodological aspects must be rigorously standardized. These include consistent image magnification, acquisition of an orthogonal plane, optimization of the insonation angle, and precise selection of the Doppler sample volume. Establishing such uniform criteria is essential to minimize operator-dependent variability and enhance measurement accuracy.

Results regarding the fetal hepatic artery Doppler assessment should be presented alongside and compared with established markers (such as DV, UA and MCA).

Future research should also focus on assessing intra- and inter-observer reproducibility, particularly for measurements obtained in early gestation, where technical challenges and anatomical variability are greater. Consistent methodology and validation studies are crucial to ensure the reliability and clinical usefulness of fetal HA Doppler evaluation in both research and clinical practice.

## 10. Conclusions

The fetal hepatic artery plays a crucial role in compensatory mechanisms aiming to maintain liver perfusion when umbilical or portal venous flow is reduced, such as through the hepatic arterial buffer response (HABR), enabling dynamic vasodilation to preserve hepatic oxygen and nutrient delivery [8,9,10].

Doppler ultrasonography has made it possible to assess fetal hepatic artery impedance in utero, providing valuable insights into fetal hemodynamics. Reduced PI or RI and increased peak PSV indicate compensatory vasodilation and have been observed in various pathological conditions such as FGR and TTTS, as well as in certain chromosomal abnormalities [8,10,12,15,16,17,18,19].

Future research should focus on standardizing measurement techniques for fetal hepatic artery Doppler assessment and validating its prognostic value. Although not yet incorporated into routine obstetric screening, fetal hepatic artery Doppler evaluation enhances understanding of fetal adaptation to hypoxia and holds promise as an adjunct tool in the assessment of fetal well-being and early detection of chromosomal abnormalities.

## Data Availability

No new data were created or analyzed in this study.

## Abbreviations

The following abbreviations are used in this manuscript:

AMP	Adenosine Monophosphate
ATP	Adenosine Triphosphate
CHD	Congenital Heart Defect
DV	Ductus Venosus
EMT	Epithelial-to-Mesenchymal Transition
EPO	Erythropoietin
FGR	Fetal Growth Restriction
HA	Hepatic Artery
HABR	Hepatic Arterial Buffer Response
HA-PI	Hepatic Artery Pulsatility Index
HSC	Hematopoietic Stem Cell
HSPC	Hematopoietic Stem and Progenitor Cell
IVC	Inferior Vena Cava
LSEC	Liver Sinusoidal Endothelial Cell
MCA	Middle Cerebral Artery
PI	Pulsatility Index
PSV	Peak Systolic Velocity
PV	Portal Vein
RI	Resistance Index
SCF	Stem Cell Factor
SEC	Sinusoidal Endothelial Cell
TGF-β	Transforming Growth Factor-β
TTTS	Twin-to-Twin Transfusion Syndrome
UA	Umbilical Artery
UV	Umbilical Vein
VEGF	Vascular Endothelial Growth Factor
VEGFR-2	Vascular Endothelial Growth Factor Receptor-2
